# PaCO2-management in the neuro-critical care of patients with subarachnoid hemorrhage

**DOI:** 10.1038/s41598-021-98462-2

**Published:** 2021-09-28

**Authors:** Marvin Darkwah Oppong, Karsten H. Wrede, Daniela Müller, Alejandro N. Santos, Laurèl Rauschenbach, Thiemo F. Dinger, Yahya Ahmadipour, Daniela Pierscianek, Mehdi Chihi, Yan Li, Cornelius Deuschl, Ulrich Sure, Ramazan Jabbarli

**Affiliations:** 1 Department of Neurosurgery and Spine Surgery, University Hospital Essen, University of Duisburg-Essen, 45147 Essen, Germany; 2 Institute for Diagnostic and Interventional Radiology and Neuroradiology, University Hospital Essen, University of Duisburg-Essen, Essen, Germany

**Keywords:** Cerebrovascular disorders, Neurovascular disorders, Stroke

## Abstract

The partial pressure of carbon dioxide (PaCO2) in the arterial blood is a strong vasomodulator affecting cerebral blood flow and the risk of cerebral edema and ischemia after acute brain injury. In turn, both complications are related to poor outcome in patients with aneurysmal subarachnoid hemorrhage (aSAH). We aimed to analyze the effect of PaCO2 levels on the course and outcome of aSAH. All patients of a single institution treated for aSAH over 13.5 years were included (n = 633). Daily PaCO2 values from arterial blood gas measurements were recorded for up to 2 weeks after ictus. The study endpoints were: delayed cerebral ischemia (DCI), need for decompressive craniectomy due to increased intracranial pressure > 20 mmHg refractory to conservative treatment and poor outcome at 6-months follow-up (modified Rankin scale > 2). By correlations with the study endpoints, clinically relevant cutoffs for the 14-days mean values for the lowest and highest daily PaCO2 levels were defined by receiver operating characteristic curve analysis. Association with the study endpoints for the identifies subgroups was analyzed using multivariate analysis. The optimal range for PaCO2 values was identified between 30 and 38 mmHg. ASAH patients with poor initial condition (WFNS 4/5) were less likely to show PaCO2 values within the range of 30–38 mmHg (*p* < 0.001, OR = 0.44). In the multivariate analysis, PaCO2 values between 30 and 38 mmHg were associated with a lower risk for decompressive craniectomy (*p* = 0.042, aOR = 0.27), DCI occurrence (*p* = 0.035; aOR = 0.50), and poor patient outcome (*p* = 0.004; aOR = 0.42). The data from this study shows an independent positive association between low normal mean PaCO2 values during the acute phase of aSAH and patients’ outcome. This effect might be attributed to the reduction of intracranial hypertension and alterations in the cerebral blood flow.

## Introduction

The partial pressure of carbon dioxide (PaCO2) in the arterial blood is a strong vasomodulator affecting the cerebral blood flow (CBF) and subsequently the intracranial pressure (ICP)^1^. This cerebrovascular reactivity seems not to be impaired in the case of aneurysmal subarachnoid hemorrhage (aSAH)^2,3^. Therefore, PaCO2 alterations might impact the risk of common complications following aneurysmal subarachnoid hemorrhage (aSAH), e.g. delayed cerebral ischemia (DCI) or elevated ICP. Both complications are related to poor outcome in patients with aSAH^4,5^.

Extreme hypocapnia (PaCO2 < 30 mmHg) has been historically used in patients with traumatic brain injury (TBI) to lower ICP but is currently abandoned due to its negative impact on outcome^6^. Current guidelines for neuro-critical care of aSAH patients include the management of cerebral vasospasm, blood pressure and increased ICP^7,8^. However, there are no recommendations on PaCO2 management and available literature is limited to some small studies. In particular, one surveillance study consisting of 6 patients revealed increased CBF and tissue oxygenation in aSAH patients with increased PaCO2 levels^9^ while Solaiman and colleagues suggested an association between prolonged hypocapnia and poor patient outcome^10^. Moreover, another trial reported on improved outcome after slightly elevated PaCO2 values during the first 24 h after ictus^11^.

In the present study, we aimed to analyze the effect of PaCO2 levels on the course and outcome of aSAH in a large institutional consecutive series.

## Methods

### Patient population

This is a retrospective study based on the institutional observational aSAH database (registered in the German clinical trial register, Unique identifier: DRKS00008749), which contains all consecutive patients with ruptured intracranial aneurysms treated at our institution between 01/2003 and 06/2016. The exclusion criteria were: (a) treatment due to a mycotic aneurysm; (b) admission > 48 h after ictus; (c) no treatment of the ruptured aneurysm; (d) hospital stay duration < 72 h after admission.

The approval of the institutional ethics committee (Ethik-Kommission, Medizinische Fakultät der Universität Duisburg-Essen, Registration number: 15-6331-BO) for this study was obtained and has been performed in accordance with the ethical standards laid down in the 1964 Declaration of Helsinki and its later amendments. The study was registered in the German clinical trial registry (DRKS, Unique identifier: DRKS00008749). All patients or their relatives gave written consent within the treatment contract before inclusion into the database.

### SAH management

All patients were initially admitted at our neurosurgical intensive care unit for at least 10 days after ictus followed by further surveillance at our intermediate care unit for at least 4 days, unless the cases with shorter survival. Acute hydrocephalus was treated by placement of an external ventricular drainage (EVD). Further conservative aSAH management was performed according to the latest guidelines^7,8^ and included oral nimodipine and euvolemia maintenance for 3 weeks. Conservative and invasive treatment of cerebral vasospasm after aSAH has been described in detail elsewhere^12^. The Bleeding source was confirmed using digital subtraction and/or computed tomography (CT)-angiography. Treatment decision was made upon interdisciplinary discussion between the interventional neuroradiologist and the vascular neurosurgeon on call. Monitoring included daily transcranial Doppler ultrasound, ICP monitoring via EVD and 3 hourly blood gas samples taken from the arterial line. Mechanical ventilation (MV) was induced for aneurysm treatment and discontinued as soon as possible depending on the patient’s pulmonary and clinical state.

All patients underwent a routine postoperative CT scan within 24 h after aneurysm treatment. Moreover, additional CT imaging was performed in cases of any neurological deterioration or prolonged impairment in the conscious state.

### Conservative and surgical ICP management

In case of sustained ICP increase > 20 mmHg, conservative treatment was initiated maintaining cerebral perfusion pressure at > 60 mmHg as follows:

1. Level 1: Infusion and catecholamines, normothermia, elevation of the head to 30°, deep sedation, osmotherapy with 20% mannitol and forced cerebrospinal fluid drainage.
2. Level 2: Relaxation, application of tromethamine, and barbiturate coma, guided by a burst-suppression-EEG pattern.

In case of persisting ICP elevation refractory to conservative treatment, decompressive craniectomy (DC) was performed. There was no specific target area for PaCO2 levels regarding ICP management.

### Data management

The digital archive and imaging were reviewed for demographic, clinical, laboratory and all radiographic parameters. To assess the value of PaCO2 for the course and outcome of aSAH, daily highest and lowest PaCO2 values during the first 14 days after ictus were recorded.

The clinical severity of aSAH was documented using the world federation of neurological surgery grading system (WFNS) scale^13^. For statistical analysis, we dichotomized patients into good (WFNS 1–3) and poor grade (WFNS grade 4–5) cases. For assessment of radiographic severity, the original Fisher scale was applied^14^, with further dichotomization into low (Fisher scale 1 and 2) and high (Fisher scale 3 and 4) grades.

Infarctions that were not related to surgical approach or intracerebral hemorrhage were documented as early infarction if present ≤ 48 h after early aneurysm occlusion and as delayed cerebral ischemia (DCI) if occurring later than 48 h after aneurysm occlusion^15^. All imaging data was reviewed by the last author (RJ) blinded at that time for any clinical information.

The total time of MV in days was documented. A MV > 7 days was defined as prolonged MV. Pneumonia was diagnosed by chest x-ray in case of pulmonary alterations and/or elevated laboratory infectious parameters. Treatment was performed according to the German guidelines for nosocomial pneumonia^16^.

The functional outcome was measured upon the routine outpatient follow-ups performed at 6 months after aSAH using the modified Rankin scale (mRS)^17^. A mRS greater than 2 was defined as poor outcome.

### Statistical analysis

The primary study endpoints were the association between PaCO2 values and: (1) the risk of poor outcome at 6 months after aSAH, (2) occurrence of DCI, and (3) the need for DC refractory to conservative treatment. PaCO2 values were additionally correlated with the patients’ baseline characteristics. SPSS 22 for Windows (IBM Corp.) was used for all statistical analysis. The significance level was set to *p* < 0.05.

According to the correlations with the primary study endpoints, clinically relevant cutoffs for the 14-days mean values for the lowest and highest daily PaCO2 levels were defined by ROC analysis. After that the whole cohort was divided into two groups:

1. Patients with PaCO2 values within the range of the ROC-defined cutoffs were referred to the “***optimal PaCO2***”-group;
2. The remaining aSAH patients with records exceeding the PaCO2 limits (lower than the lowest and/or higher than the highest cutoff) were referred to the “***non-optimal PaCO2***”-group.

The associations between the allocation to the PaCO2-related groups and the study endpoints were assessed in a multivariable manner using binary logistic regression analysis for the study endpoints adjusted for endpoint-relevant confounders. To analyze of the need for DC in cases refractory to conservative ICP treatment, the cases with DC performed at admission prior to the initiation of conservative ICP treatment (n = 145) were excluded. Missing data was managed utilizing multiple imputations.

## Results

### Patient population

**Figure 1:**
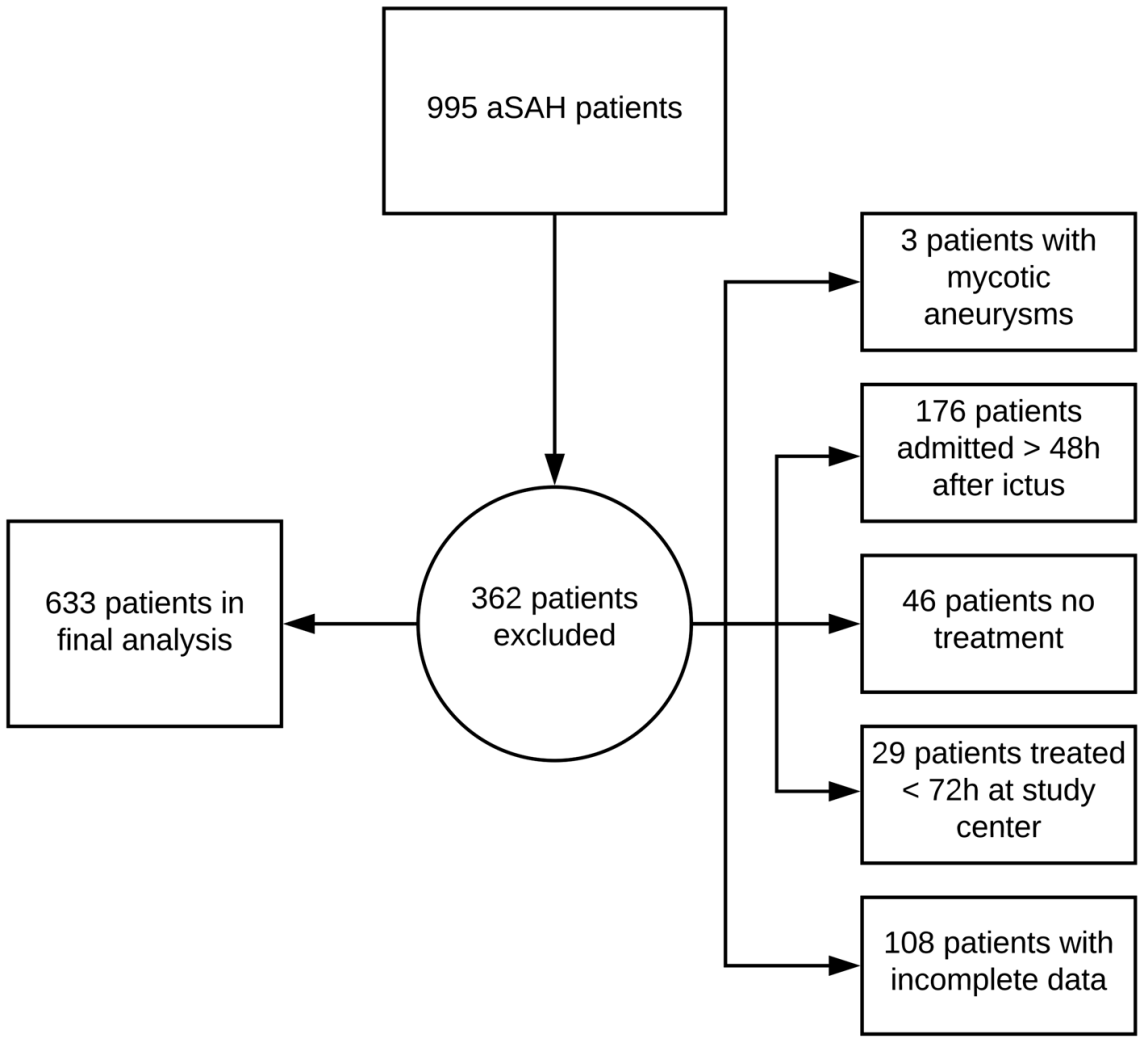
Flow-Chart of recruitment process for this study.

A total of 633 patients were eligible for this study (see Fig. 1). The majority of patients were females (n = 422; 66.7%) and presented with severe aSAH on the initial CT scan (n = 538; 91.2%). About half of the patients were at WFNS grade 4–5 at admission (n = 300; 47.4%). DCI occurred in one of four patients (n = 153; 24.2%) and 49.5% of all cases (n = 311) revealed poor outcome at 6-month follow-up. See also Table 1 in the supplemental material for a detailed overview of the cohort characteristics.

**Table 1:** Overview of lowest/highest mean PaCO2 value distribution among the different endpoints.

Endpoint	Mean lowest PaCO2 (mmHg)		Mean highest PaCO2 (mmHg)	
Poor outcome (*yes/no*)	31.57 ± 4.55	32.04 ± 4.79	40.20 ± 3.75	38.85 ± 4.09
DCI (*yes/no*)	31.16 ± 3.84	32.00 ± 4.89	40.02 ± 3.70	39.33 ± 4.06
DC due to refractory ICP increase (*yes/no*)	30.84 ± 2.87	32.04 ± 5.27	40.12 ± 3.56	39.22 ± 4.03

### Identification of clinically relevant lowest/highest PaCO2 cutoffs and allocation of the patients to the PaCO2-related groups

**Figure 2:**
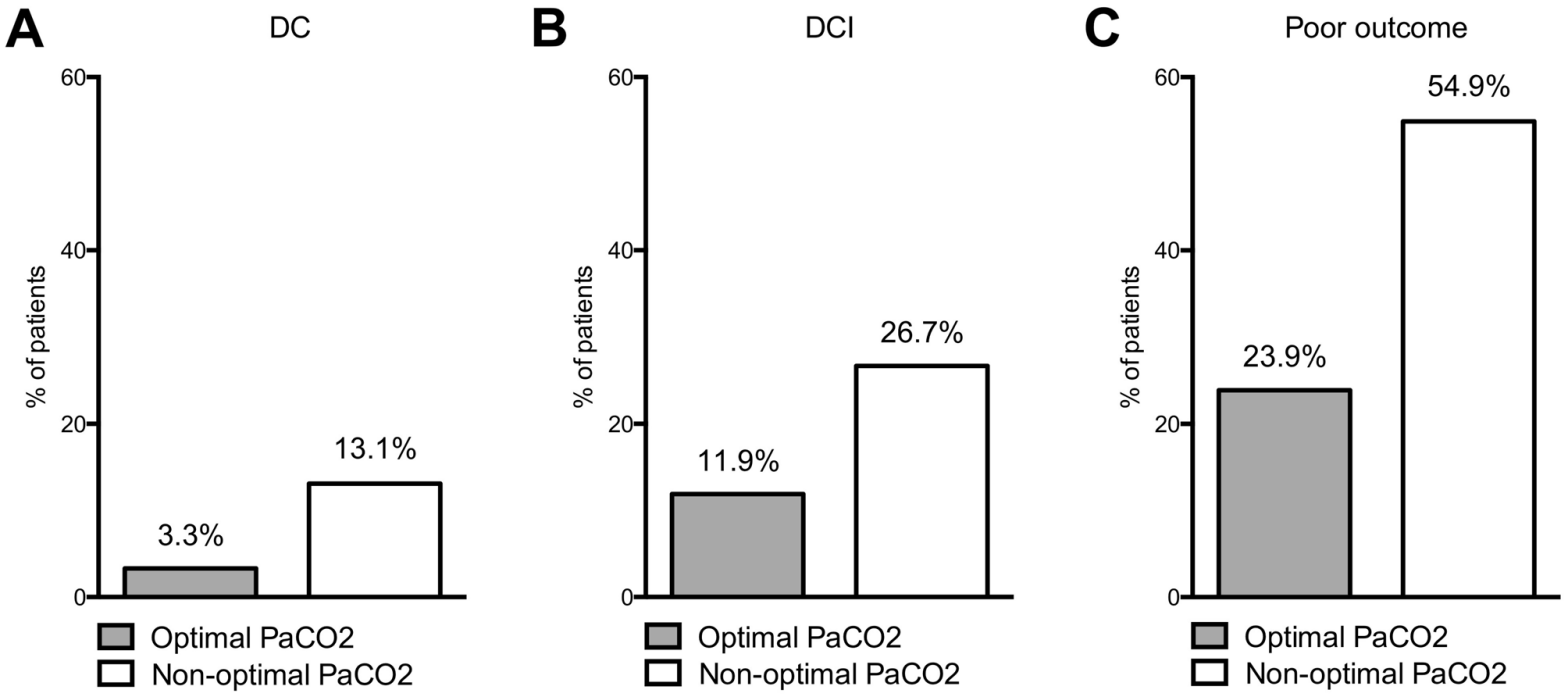
Distribution of risk for (**A**) DC due to refractory ICP increase, (**B**) DCI and (**C**) poor outcome between the optimal PaCO2 and the non-optimal PaCO2 group.

In accordance with the ROC analysis investigating the 14-days lowest/highest mean PaCO2 levels and the study endpoints, the clinically relevant cutoff for the lowest 14-days mean PaCO2 was set at 30 mmHg, and the cutoff for the highest 14-days mean PaCO2 at 38 mmHg. Accordingly, aSAH patients with PaCO2 values within the above-mentioned ranges (n = 109) were referred to the “***optimal PaCO2***” group, whereat the remaining individuals (n = 524) were included in the “***non-optimal PaCO2***” group. See also Figs. 1, 2 and Table 2 in the supplemental material. A detailed listing of mean PaCO2 values for the different endpoints is given in Table 1.

**Table 2:** Statistical analysis of demographic and clinical baseline characteristics for optimal and non-optimal PaCO2 groups.

Parameter	Optimal PaCO2	Non optimal PaCO2	*p*	OR	95% CI
	%/mean ± SD	%/mean ± SD			
Age (years)	52.7 ± 14.0	54.9 ± 13.6	0.136	–	–
Sex female	67.9%	66.4%	0.766	1.07	0.69–1.66
WFNS 4/5	31.2%	50.8%	** < 0.001**	0.44	0.28–0.68
Fisher 3/4	90.4%	91.3%	0.777	0.90	0.42–1.91
Treatment clipping	37.6%	42.0%	0.399	0.83	0.55–1.27

Statistically significant *p*-values are marked bold.

### PaCO2-groups and baseline characteristics

ASAH individuals with poor initial clinical condition (WFNS grade 4–5) were less likely to show PaCO2 values within the optimal range (*p* < 0.001; odds ratio [OR] = 0.44; 95% confidence interval [95%CI] 0.28–0.68). Other baseline demographic and clinical characteristics of the patients showed no significant associations with the PaCO2-groups (Table 2).

### Surgical ICP management

Individuals in the “optimal PaCO2” group showed less frequently an ICP increase unresponsive to conservative therapy. Accordingly, this patient group was less likely to require DC (*p* = 0.042; adjusted [a]OR = 0.27; 95%CI 0.08–0.96, see Table 3 and Fig. 2A).

**Table 3:** Multivariate analysis of factors affecting the need for DC.

Parameter	*p*	aOR	95% CI
WFNS 4/5	0.158	1.64	0.83–3.27
Fisher 3/4	0.715	1.31	0.31–5.50
Age (> 65 years)	**0.017**	0.30	0.11–0.81
Conservative ICP therapy level 1	** < 0.001**	41.3	9.79–174.23
Conservative ICP therapy level 2	0.357	1.72	0.54–5.45
Optimal PaCo2 (30–38 mmHg)	**0.042**	0.27	0.08–0.96

Statistically significant *p*-values are marked bold.

### DCI and vasospasm risk

The DCI’s overall occurrence rate was significantly reduced in the “optimal PaCO2” group (Fig. 2B). This effect of PaCO2 on DCI occurrence was independent of clinical/radiographic aSAH severity, age, vasospasm, pneumonia and prolonged MV (*p* = 0.035; aOR = 0.50; 95%CI 0.26–0.95; Table 4).

**Table 4:** Multivariate analysis of factors impacting the occurrence of DCI and poor outcome.

Parameter	*p*	aOR	95% CI
WFNS 4/5	**0.007**	1.80	1.17–2.77
Fisher 3/4	0.112	2.51	0.08–7.86
Age (> 65 years)	0.104	1.46	0.92–2.32
Vasospasm	** < 0.001**	2.89	1.97–4.46
Pneumonia	**0.006**	1.87	1.20–2.93
Prolonged MV(> 7 days)	0.417	1.20	0.77–1.86
Optimal PaCo2 (30–38 mmHg)	**0.035**	0.50	0.26–0.95

Statistically significant *p*-values are marked bold.

### Functional outcome

Patients in the “optimal PaCO2” group showed significantly reduced risk for a poor outcome at 6-month follow-up in multivariate analysis: *p* = 0.004; aOR = 0.42; 95%CI 0.23–0.76 (see Table 5 and Fig. 2C).

**Table 5:** Multivariate analysis of factors impacting the occurrence of poor outcome.

Parameter	*p*	aOR	95% CI
WFNS 4/5	** < 0.001**	3.27	2.14–5.00
Fisher 3/4	**0.011**	3.61	1.35–9.65
Age (> 65 years)	** < 0.001**	3.30	2.00–5.45
DCI	** < 0.001**	6.45	3.78–11.00
Pneumonia	**0.006**	2.10	1.24–3.57
Prolonged MV (> 7 days)	** > 0.001**	2.78	1.83–4.23
Optimal PaCO2 (30–38 mmHg)	**0.004**	0.42	0.23–0.76

Statistically significant *p*-values are marked bold.

## Discussion

This study aimed to evaluate the influence of PaCO2 levels on major complications (like occurrence of DCI and intractable ICP increase) and functional outcome of aSAH. Therefore, we defined an optimal PaCO2 window between moderate hypocapnia (≥ 30 mmHg) and slightly elevated normocapnia (≤ 38 mmHg). ASAH individuals with PaCO2 values during the first 2 weeks within this range were at lower risk of DCI, DC due to conservatively intractable ICP increase and poor outcome 6 months after aSAH.

### PaCO2 and ICP elevations refractory to conservative therapy

In case of malignant infarction, DC is a common neurosurgical treatment option for sustained ICP increase refractory to conservative management^18^. In contrast to stroke, no clear guidelines regarding the indication and timing of DC exist in the setting of aSAH^19^. Patients that develop sustained ICP increase necessitating DC often face a poor outcome^19–21^. Therefore, early prevention of such intractable ICP increase is of paramount importance for aSAH patients’ outcome.

It is generally acknowledged that the decreasing of PaCO2 leads to lower ICP values^22^ and the pathophysiological background of this effect is related to arteriolar vasoconstriction, especially in the pial and cortical vessels. This mechanism reduces CBF and is believed to subsequently reduce ICP by reducing the development of cerebral edema^23,24^. Historically, extreme hypocapnia (< 30 mmHg) was commonly used to lower ICP in patients with TBI^6^. However, this practice was later abandoned due to poor functional outcome of individuals treated with PaCO2 < 30 mmHg^6^. Of note, the ICP-lowering effect of PaCO2 might be present also at moderate hypocapnia^23^. In our cohort, individuals with consistently low-normal PaCO2 values ranging between 30 and 38 mmHg were at lower risk for ICP increase unresponsive to conservative therapy than the remaining aSAH cohort. Therefore, this PaCO2 management might become a useful component of conservative ICP treatment in aSAH.

### PaCO2 and DCI risk

The occurrence of DCI has a paramount impact on the course and outcome of aSAH^25^. DCI’s exact pathophysiology in aSAH has not yet been completely understood, but multi-factorial genesis of DCI is supposed^26^. Cerebral vasospasm is one major mechanism of DCI and aSAH management includes nimodipine application, induced hypertension and angiographic vasospasmolysis^25,27^. Despite cerebral vasospasm, inflammatory factors, micro-thrombosis and cortical spreading depolarization seem to play a role in DCI development^4,28–31^.

Strong alterations of PaCO2 in both directions might also contribute to DCI risk after aSAH. On one side, hypercapnia is associated with ICP increase and subsequent decrease of cerebral perfusion pressure and CBF. On the other side, hypocapnia also results in CBF decline due to vasoconstriction. At the same time, induced hypertension initiated in patients with cerebral vasospasm might alleviate the vasoconstrictory effect of hypocapnia on CBF. Therefore, the negative clinical impact of hypocapnia observed in TBI cohorts is not to be expected to the same extent in aSAH. Instead, moderate hypocapnia and induced hypertension potentially present an optimal framework condition for the prevention of DCI. This constellation might explain our results showing that aSAH patients with PaCO2 values ranging between 30 and 38 mmHg were at lower DCI risk. This independent association between PaCO2 and DCI risk encourages further research of the potential link between PaCO2, CBF and DCI in aSAH patients.

### PaCO2 and outcome following aSAH

Our data underlines that moderately decreased PaCO2 independently reduces the risk of poor outcome following aSAH. In TBI and cerebrovascular accidents, aggressive hypocapnia has been associated with poor outcome and mortality^32–34^. At least for TBI, the critical cut-off was reported at the values between 27 and 32 mmHg^33,34^. Also no relevant advantage could be shown for extreme hypocapnia (30 mmHg) in TBI treatment^6^. Comparable results exist for stroke^35^. Interestingly, in a subgroup analysis excluding major vessel occlusion and mainly including hemorrhagic stroke, hypocapnia showed a tendency to lower mortality and better outcome^35^. Data from aSAH are very sparse and suggest poorer outcome in patients regularly having PaCO2 levels below 35 mmHg^10^. This discrepancy to our results might be related to the fact that the “ < 35 mmHg PaCO2” group in this study^10^ also included individuals with PaCO2 values < 30 mmHg. Recent published data suggests that PaCO2 values regularly reaching high normal values (> 40 mmHg) are harmful for aSAH patients^36^.

In summary, the positive effect of “low-normal PaCO2” management during the first 2 weeks after aneurysm rupture on the risk of DCI, intractable ICP increase and poor outcome after aSAH might speak for the implementation of specific PaCO2 target area in future guidelines for the management of aSAH patients requiring MV, provided the confirmation of our results in a prospective controlled trial.

## Limitations

This studys main drawback is its retrospective design, resulting in lower accuracy and consistency of data than in comparable prospective collected data sets. This disadvantage might be partly outweighed by the cohort’s size, strict exclusion criteria and robust statistical assessment. Our data does not allow a direct correlation between PaCO2 level and corresponding ICP. This connection was only made indirectly by using the clinical surrogate parameters of increased ICP, i.e. conservative and surgical ICP therapy. Furthermore, this study does not exclusively include patients that were ventilated for the whole surveillance period. To address this potential risk of bias, the results were corrected for the duration of ventilation.

## Conclusions

Our data suggests that patients with aSAH might profit from low-normal PaCO2 values during the first weeks after aneurysm rupture. This effect is highlighted by reducing DCI and intracranial hypertension, resulting in lower rate of poor outcome in aSAH individuals with PaCO2 values ranging between 30 and 38 mmHg in our cohort. The results from this study urge for a prospective evaluation of the clinical benefit of low-normal PaCO2 values in the treatment of aSAH.

## Supplementary Information

Supplementary Information 1.

## References

[1] Ainslie PN, Duffin J (2009). Integration of cerebrovascular CO2 reactivity and chemoreflex control of breathing: Mechanisms of regulation, measurement, and interpretation. Am. J. Physiol. Regul. Integr. Comp. Physiol..

[2] Romner B (1991). Simultaneous transcranial Doppler sonography and cerebral blood flow measurements of cerebrovascular CO2-reactivity in patients with aneurysmal subarachnoid haemorrhage. Br. J. Neurosurg..

[3] Diringer MN, Kirsch JR, Hanley DF, Traystman RJ (1993). Altered cerebrovascular CO2 reactivity following subarachnoid hemorrhage in cats. J. Neurosurg..

[4] DarkwahOppong M (2018). Post-treatment antiplatelet therapy reduces risk for delayed cerebral ischemia due to aneurysmal subarachnoid hemorrhage. Neurosurgery.

[5] Mohme M (2017). Irreversible total loss of brain function and organ donation in patients with aneurysmal subarachnoid hemorrhage. World Neurosurg..

[6] Muizelaar JP (1991). Adverse effects of prolonged hyperventilation in patients with severe head injury: A randomized clinical trial. J. Neurosurg..

[7] Connolly ES (2012). Guidelines for the management of aneurysmal subarachnoid hemorrhage: A guideline for healthcare professionals from the American Heart Association/American Stroke Association. Stroke; J. Cereb. Circ..

[8] Steiner T (2013). European stroke organization guidelines for the management of intracranial aneurysms and subarachnoid haemorrhage. Cerebrovasc. Dis. (Basel, Switz.).

[9] Westermaier T (2014). Controlled transient hypercapnia: A novel approach for the treatment of delayed cerebral ischemia after subarachnoid hemorrhage?. J. Neurosurg..

[10] Solaiman O, Singh JM (2013). Hypocapnia in aneurysmal subarachnoid hemorrhage: Incidence and association with poor clinical outcomes. J. Neurosurg. Anesthesiol..

[11] Lång M (2016). Early moderate hyperoxemia does not predict outcome after aneurysmal subarachnoid hemorrhage. Neurosurgery.

[12] Jabbarli R (2019). Endovascular treatment of cerebral vasospasm after subarachnoid hemorrhage: More is more. Neurology.

[13] Teasdale GM (1988). A universal subarachnoid hemorrhage scale: Report of a committee of the world federation of neurosurgical societies. J. Neurol. Neurosurg. Psychiatr..

[14] Fisher CM, Kistler JP, Davis JM (1980). Relation of cerebral vasospasm to subarachnoid hemorrhage visualized by computerized tomographic scanning. Neurosurgery.

[15] Vergouwen MD (2010). Definition of delayed cerebral ischemia after aneurysmal subarachnoid hemorrhage as an outcome event in clinical trials and observational studies: Proposal of a multidisciplinary research group. Stroke; J. Cereb. Circ..

[16] Lorenz J (2003). Nosocomial pneumonia: Prevention, diagnosis, treatment. Pneumol. (Stuttg., Ger.).

[17] van Swieten JC, Koudstaal PJ, Visser MC, Schouten HJ, van Gijn J (1988). Interobserver agreement for the assessment of handicap in stroke patients. Stroke; J. Cereb. Circ..

[18] Warner JJ, Harrington RA, Sacco RL, Elkind MSV (2019). Guidelines for the early management of patients with acute ischemic stroke: 2019 update to the 2018 guidelines for the early management of acute ischemic stroke. Stroke; J. Cereb. Circ..

[19] DarkwahOppong M (2020). Decompressive craniectomy in aneurysmal subarachnoid hemorrhage: Who and when?—A systematic review and meta-analysis. Clin. Neurol. Neurosurg..

[20] Jabbarli R (2017). Time is brain! Analysis of 245 cases with decompressive craniectomy due to subarachnoid hemorrhage. World Neurosurg..

[21] Jabbarli R (2020). The pressure score to predict decompressive craniectomy after aneurysmal subarachnoid haemorrhage. Brain Commun..

[22] Raichle ME, Plum F (1972). Hyperventilation and cerebral blood flow. Stroke; J. Cereb. Circ..

[23] Sadoshima S (1980). Response of cortical and pial arteries to changes of arterial CO2 tension in rats—A morphometric study. Brain Res..

[24] Tuor UI, Farrar JK (1984). Pial vessel caliber and cerebral blood flow during hemorrhage and hypercapnia in the rabbit. Am. J. Physiol..

[25] Francoeur CL, Mayer SA (2016). Management of delayed cerebral ischemia after subarachnoid hemorrhage. Crit. Care (Lond., Engl.).

[26] Geraghty JR, Testai FD (2017). Delayed cerebral ischemia after subarachnoid hemorrhage: Beyond vasospasm and towards a multifactorial pathophysiology. Curr. Atheroscler. Rep..

[27] Etminan N, Vergouwen MD, Ilodigwe D, Macdonald RL (2011). Effect of pharmaceutical treatment on vasospasm, delayed cerebral ischemia, and clinical outcome in patients with aneurysmal subarachnoid hemorrhage: A systematic review and meta-analysis. J. Cereb. Blood Flow Metabol.: Off. J. Int. Soc. Cereb. Blood Flow Metabol..

[28] Frontera JA (2017). The role of platelet activation and inflammation in early brain injury following subarachnoid hemorrhage. Neurocrit. Care.

[29] McBride DW, Blackburn SL, Peeyush KT, Matsumura K, Zhang JH (2017). The role of thromboinflammation in delayed cerebral ischemia after subarachnoid hemorrhage. Front. Neurol..

[30] Foreman B (2016). The pathophysiology of delayed cerebral ischemia. J. Clin. Neurophysiol.: Off. Publ. Am. Electroencephalogr. Soc..

[31] Jabbarli R (2018). Laboratory biomarkers of delayed cerebral ischemia after subarachnoid hemorrhage: A systematic review. Neurosurg. Rev..

[32] Rout MW, Lane DJ, Wollner L (1971). Prognosis in acute cerebrovascular accidents in relation to respiratory pattern and blood gas tensions. BMJ.

[33] Davis, D. P. *et al.* The impact of hypoxia and hyperventilation on outcome after paramedic rapid sequence intubation of severely head-injured patients. *J. Trauma* **57**, 1–8; discussion 8–10. 10.1097/01.ta.0000135503.71684.c8 (2004). 10.1097/01.ta.0000135503.71684.c8 15284540

[34] Davis DP (2006). Early ventilation and outcome in patients with moderate to severe traumatic brain injury. Crit. Care Med..

[35] Christensen MS (1973). Cerebral apoplexy (stroke) treated with or without prolonged artificial hyperventilation. 1. Cerebral circulation, clinical course, and cause of death. Stroke; J. Cereb. Circ..

[36] Reiff T, Barthel O, Schönenberger S, Mundiyanapurath S (2020). High-normal P(a)CO(2) values might be associated with worse outcome in patients with subarachnoid hemorrhage—A retrospective cohort study. BMC Neurol..

