# Supplementary material for: PaCO2-management in the neuro-critical care of patients with subarachnoid hemorrhage
Source: Sci Rep. 2021 Sep 28;11:19191. doi: 10.1038/s41598-021-98462-2 (PMC8478930; doi:10.1038/s41598-021-98462-2)
Supplement: Supplementary file 1 — Supplementary Information 1. [file 41598_2021_98462_MOESM1_ESM.docx]

**SUPPLEMENTS**

Figure 1: ROC analysis utilized for cut-off definition regarding daily mean highest PaCO2.

**
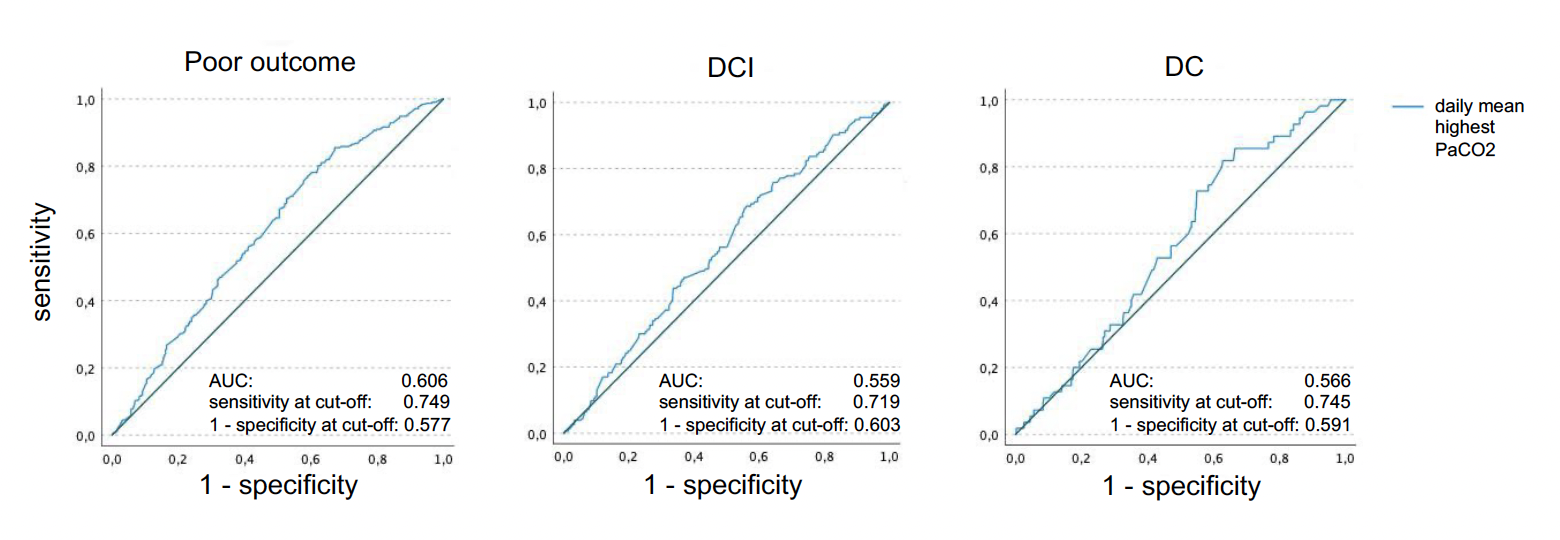
**

Figure 2: ROC analysis utilized for cut-off definition regarding daily mean lowest PaCO2.


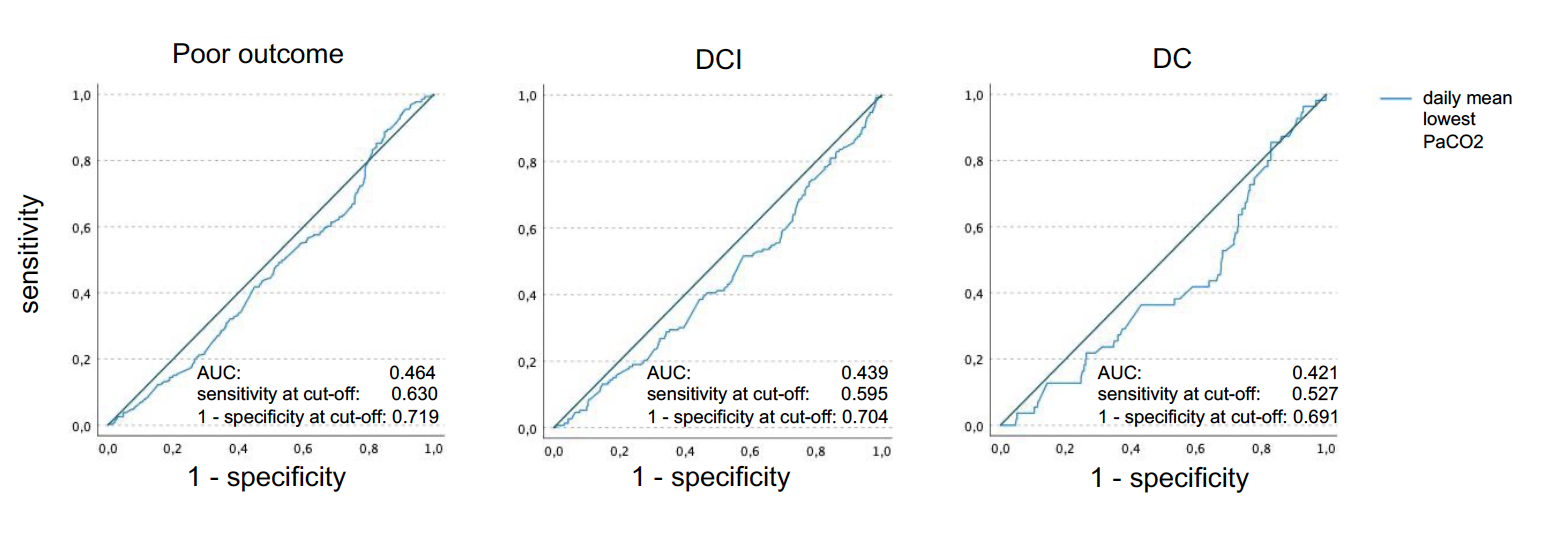


| **Parameter** |  | **%/mean±SD** |
| --- | --- | --- |
| Female sex |  | 66.7% |
| Age (years) |  | 54.57±13.66 |
| WFNS 4/5 |  | 47.4% |
| Fisher 3/4 * |  | 91.2% |
| Acute Hydrocephalus |  | 78.2% |
| Treatment clipping |  | 41.2% |
| *Aneurysm location* |  |  |
|  | MCA | 24.0% |
|  | ICA | 10.7% |
|  | pACA | 35.1% |
|  | dACA | 3.3% |
|  | Posterior circulation | 26.9% |
| *Pulmonary complications* |  |  |
|  | Pneumonia † | 23.7% |
|  | Prolonged MV‡ | 52.6% |
| *Ventilation/Blood gas* |  |  |
|  | Time of MV (days) ‡ | 8.1 ± 6.0 |
|  | Mean daily highest PaCO2(mmHg) | 39.50 ± 3.98 |
|  | Mean daily highest PaCO2(mmHg) | 31.79 ± 4.66 |
| *Infarction* |  |  |
|  | Early# | 30.9% |
|  | DCI# | 24.2% |
| Vasospasm |  | 22.7% |
| *DC* |  |  |
|  | DC before conservative treatment | 22.9% |
|  | DC due to refractory ICP increase | 11.3% |
| Conservative ICP Therapy Level 1** |  | 46.3% |
| Conservative ICP Therapy Level 2** |  | 3.0% |
| Poor Outcome at 6-month follow-up *** |  | 49.5% |
| In hospital mortality |  | 14.2% |
| Optimal PaCO2 (30-38 mmHg) |  | 17.2% |
| * Data missing for 43 patients  † Data missing for 29 patients  ‡ Data missing for 4 patients  # Data missing for 1 patient  ** Data missing for 3 patients  ***Data missing for 5 patients | | |

Table 1: Overview of baseline characteristics of the study cohort.

| ***Parameter*** | **Mean lowest PaCO2 <30mmHg** | **Optimal PaCO2 (30-38mmHg)** | **Mean highest PaCO2 >38mmHg** |
| --- | --- | --- | --- |
| Number of patients (n)* | 190 | 109 | 401 |
| Age (years/mean±SD) | 53.16±12.85 | 52.74±14.00 | 55.51±13.72 |
| Prolonged MV(%) | 54.5% | 26.6% | 61.5% |
| DCI (%) | 30.7% | 11.9% | 27.5% |
| Poor outcome (%) | 56.7% | 23.9% | 56.0% |
| DC due to refractory ICP increase (%) | 16.8% | 3.3% | 13.7% |
| ICU > 10 days (%) | 49.7% | 22.9% | 55.2% |
| Mean lowest PaCO2 (mmHg/mean±SD) | 28.24±1.49 | 31.54±1.48 | - |
| Mean highest PaCO2 (mmHg/mean±SD) | - | 36.33±1.38 | 41.70±3.01 |
| *67 patients exceeded the cut-offs in both groups (mean daily lowest and mean highest PaCO2 ) | | | |

Table 2: Overview over distribution of patients exceeding the mean daily cut-offs volumes for mean lowest and mean highest PaCO2 in comparison with the optimal PaCO2 group regarding endpoints, prolonged MV and absolute PaCO2 values.

| ***Parameter*** | **p** | **aOR** | **95% CI** |
| --- | --- | --- | --- |
| Total time of MV (completely controlled and supported) (days) | 0.003 | 0.86 | 0.78 - 0.95 |
| Total time of completely controlled MV (days) | 0.672 | 0.91 | 0.91 - 1.15 |

Table 3.: Multivariate analysis of influence of mechanical ventilation mode on the occurrence of “optimal PaCO2” values.
